# A systems genomics approach to uncover the molecular properties of cancer genes

**DOI:** 10.1038/s41598-020-75400-2

**Published:** 2020-10-27

**Authors:** Felix Grassmann, Yudi Pawitan, Kamila Czene

**Affiliations:** Department of Medical Epidemiology and Biostatistics, Karolinska Institutet, Nobels väg 12A, 171 65 Stockholm, Sweden

**Keywords:** Cancer genomics, Data integration, Genome informatics, Cancer genomics, Functional genomics, Gene expression, Evolutionary biology

## Abstract

Genes involved in cancer are under constant evolutionary pressure, potentially resulting in diverse molecular properties. In this study, we explore 23 *omic* features from publicly available databases to define the molecular profile of different classes of cancer genes. Cancer genes were grouped according to mutational landscape (germline and somatically mutated genes), role in cancer initiation (cancer driver genes) or cancer survival (survival genes), as well as being implicated by genome-wide association studies (GWAS genes). For each gene, we also computed feature scores based on all omic features, effectively summarizing how closely a gene resembles cancer genes of the respective class. In general, cancer genes are longer, have a lower GC content, have more isoforms with shorter exons, are expressed in more tissues and have more transcription factor binding sites than non-cancer genes. We found that germline genes more closely resemble single tissue GWAS genes while somatic genes are more similar to pleiotropic cancer GWAS genes. As a proof-of-principle, we utilized aggregated feature scores to prioritize genes in breast cancer GWAS loci and found that top ranking genes were enriched in cancer related pathways. In conclusion, we have identified multiple omic features associated with different classes of cancer genes, which can assist prioritization of genes in cancer gene discovery.

## Introduction

One of the main challenges in cancer genetics is the identification of genes involved in cancer risk and prognosis and to characterise their molecular function in health and disease. While our knowledge on the function of a small number cancer genes is quite substantial, little is known about the role of most cancer genes due to the identification of an ever-increasing number of cancer genes, which outpaces functional characterization^1,2^. However, the presently large number of known or suspected cancer genes also has the potential to enable the broad and comprehensive characterization of those genes to identify the molecular and evolutionary patterns underlying either all cancer genes or specific classes thereof.

Generally, cancer genes can be divided into several distinct classes according to their involvement in cancer risk or prognosis as well as according to their molecular function, mode of inheritance and mutational landscape^3,4^. Recent large-scale sequencing efforts in tumour cells and tissue provided a comprehensive assessment of aberrations in somatic cancer genes and their influence on different hallmarks of cancer^5^. Further advances in bioinformatic and experimental approaches allowed to leverage those large datasets which led to the description of new cancer driver genes^3,6^. While somatically acquired mutations in cancer genes are important for the initiation and progression of tumours, they also seem to play an important role in cancer specific survival^7,8^. Furthermore, the expression levels of thousands of genes are involved in survival from cancer, often with pleiotropic and contrasting effects on different types of cancer^9,10^.

In addition to genes mainly involved in cancer prognosis and aggressiveness, an ever-expanding number of genes are identified which play a role in cancer risk and thus are mostly responsible in disease processes before diagnosis. The occurrence of tumours is heritable, with heritability estimates ranging from 9 to 57%^11^. A sizable portion of familial cancer risk can be attributed to rare mutations in germline cancer genes^4^, although only around a hundred germline cancer genes have been identified so far^12^ most of which are tumour suppressors^13^. Conversely, a significant part of the remaining heritability is likely attributable to common cancer risk increasing variants^14^, which are routinely identified by genome-wide association studies (GWAS). In contrast to germline mutations which are usually located within the coding region of a gene and thus directly implicate the responsible gene, common cancer variants do not directly implicate a particular gene target or even molecular pathway due to extensive linkage between variants. Consequently, an association signal may span a large region in the genome covering many potentially relevant genes, thus posing an obstacle for future in-depth functional characterisation of the casual gene(s). Therefore, in order to prioritize the most likely cancer gene within a region, multiple algorithms have been developed which implicate genes according to mutational load, molecular function, involvement in specific pathways or expression^15–20^. Alternatively, understanding the molecular characteristics of typical cancer genes promises to allow the prioritization of genes within those regions by implicating those genes which most closely resemble other typical cancer genes.

Therefore, to further characterize the molecular properties of cancer genes, we systematically investigate the multiple *omic* features of different classes of cancer genes. We then aggregate the effect of those features to rank genes within breast cancer GWAS regions and perform pathway enrichment on genes to illustrate the utility of our findings.

## Methods

### Cancer gene definition

658 genes frequently mutated in tumours (somatic genes) as well as 107 genes that often harbour rare mutations causing hereditary cancer (germline genes) located were extracted from the Catalogue of Somatic Mutations in Cancer (COSMIC, URL: https://cancer.sanger.ac.uk/cosmic) Cancer Gene Census (CGC)^5^. We considered both, Tier 1 and Tier 2 genes (i.e. genes with strong indications of a role in cancer) in our analyses as cancer genes in order to maximize the number of observations (i.e. genes) in each cancer class. Furthermore, we included 294 genes recently described to harbour cancer driver mutations (driver genes)^2^. Several germline, somatic and driver genes were identified to be part of multiple gene sets and we made the following exclusions: 64 genes which were listed as both germline and somatic were excluded from both lists to create a clean somatic and germline gene set, respectively. In addition, we excluded 188 genes from the driver gene list since they are also considered either somatic or germline genes as well as 137 genes from the somatic gene list which are driver genes.

The associations of gene expression with cancer survival in The Cancer Genome Atlas (TCGA, URL: https://portal.gdc.cancer.gov/) were extracted from GEPIA (Gene Expression Profiling Interactive Analysis, URL: https://gepia.cancer-pku.cn/)10. We considered 1719 genes which are significantly associated with survival (false discovery rate < 0.01) in at least two datasets to be cancer survival genes to reduce the number of false positives and to include genes with a strong evidence for their involvement in cancer survival. We also separately investigated cancer survival genes whose expression is significantly positively (i.e. hazard ratio greater than one) or negatively (hazard ratio smaller than one) correlated to survival from cancer. 451 survival genes which are also deemed germline, somatic or driver cancer genes or which are located in cancer GWAS loci (see below) were excluded from their respective analyses to create a clean set of genes involved in cancer survival.

Finally, we included genes within cancer loci identified by genome-wide association studies. In order to account for the large number of associations tested in GWAS, the accepted threshold for a significant association with a trait (i.e. genome-wide significance) is 5.00 × 10^−08^. Therefore, we extracted all genome-wide significant variants associated with any cancer from the GWAS catalogue^21^ (accession date: 2018-06-25, URL: https://www.ebi.ac.uk/gwas/) and removed variants associated with cancer severity/aggressiveness, survival and other non-disease-risk associated outcomes. Furthermore, we only considered association signals primarily identified in Europeans.

We grouped the cancer association signals into 20 clusters according to their respective tissue: breast, skin, colorectal, ovarian, uterus, testicular, thyroid, oesophageal, lung, renal, prostate, pancreas, oral, bladder, cervix, haematological and central nervous cancer as well as meningioma, sarcoma, and uveal melanoma. We separately investigated genes in GWAS regions with cancer association signals for only one tissues (GWAS genes) or with multiple signals from cancers of different tissues or types of cancer (pleiotropic GWAS genes).

The lower and upper boundaries of a cancer GWAS locus were defined by the most distant variants in moderate linkage disequilibrium to the index GWAS variant (D’ < 0.5). We then merged GWAS loci with genomic overlap and considered a total of 4075 genes within those loci as cancer genes in case the transcription start site (TSS) or the transcription terminator of the longest transcript is located within the locus boundaries. In the analysis of GWAS cancer genes, we excluded 1702 genes which were considered germline, somatic, driver or survival cancer genes.

### Extraction of features

All genomic coordinates mentioned in the manuscript are based on hg19, since various databases reported their features based on the hg19 genome assembly at the time of analysis. Liftover from other builds was performed with the *rtracklayer* library (version 1.42.1) as implemented in R (version 3.5.1, URL: https://www.R-project.org)^22^. The necessary liftover chain files were downloaded from UCSC Genome browser (https://hgdownload.cse.ucsc.edu/goldenpath/hg19/liftOver/). We used biomaRt^23^ (version 2.38.0) implemented in R to extract genomic features from the ENSEMBL database. In particular, we extracted the size of the gene body (i.e. genomic distance between transcription start site and transcription terminator of the longest transcript) and the number of isoforms and exons of each protein coding gene. Furthermore, we documented the percent GC content of each gene within the gene body and recorded the mean length of the 3′ and 5′ UTR as well as the average size of all exons of all isoforms.

In addition, we calculated the number of orthologues within the family Hominidae to address recent evolutionary conservation as well as the number of paralogues within the human genome to investigate the presence of potentially redundant gene copies.

In order to investigate the linkage disequilibrium structure within cancer genes, we computed the average and standard deviation of the LD Scores derived from European populations^10^ of all variants within the gene body.

Genes which have fewer mutations than expected are considered essential and mutation intolerant. The degree of intolerance can be expressed as a Z Score of intolerance. For each coding gene, we extracted the Z scores for synonymous, non-synonymous as well the loss of function mutations from gnomAD^10^.

Next, we computed multiple measures to summarize gene expression and regulation of cancer genes across all tissues included in GTEx^24^. We computed the mean and standard deviation of gene expression values (expressed as Transcripts Per Kilobase Million, TPM) across all 44 tissues and also counted the number of tissues a gene is expressed in (i.e. TPM > 1). Furthermore, we extracted the number of unique variants across all tissues that are significantly correlated to gene expression (expression quantitative trait loci, eQTL) for each gene.

We also calculated the number of transcription factor (TF) binding sites (identified by ChIPSeq experiments) reported in the Gene Transcription Regulation Database (GTRD)^25^ either within the gene body, within the promotor (i.e. 1000 bp up- and downstream of the TSS of a gene) or within the distal region of a gene (within 100,000 bp up- and downstream of the TSS or transcription terminator, respectively). The number of TF sites in the gene body as well as in the distal region was then normalized/divided by the gene length. Transcription factors were also grouped into canonical classes according to Lambert et al.^26^ and we estimated the association of TF binding sites in the promotor from individual classes with cancer genes.

Finally, for each gene, we computed the total number of common post-translational protein modifications reported by dbPTM^27^ as well as the occurrence of individual modifications reported in at least 10 genes.

### Computation of scores from cancer features and statistical analyses

All analyses were restricted to protein coding genes outside of the highly pleiotropic *MHC* region on chromosome 6 (hg19 coordinates: 25,477,797 bp–33,448,354 bp). All features were scaled to have a mean of zero and a standard deviation of one in order to account for differences in the scale of the underlying data. The association of the features with the binary cancer gene status was evaluated with logistic regression as implemented in R, since logistic regression does not assume a specific distribution of the predictor variables, many of which are potentially not normally distributed. The outcome (dependent variable) of those models was the binary assignment to either the respective cancer class or to the background gene set. The exposures in the models were the different omic features and the effect sizes were visualized as a correlation plot with the *corrplot* function from the *corrplot* package (version 0.84). The association of genomic features with GWAS cancer genes as adjusted for the number of genotyping probes from genotyping chips within each gene body to account for potential confounding effects due to coverage.

Similar to the computation of genetic risk scores^28,29^, we computed genomic cancer feature scores of all cancer gene classes for each gene. We multiplied (weighted) the value of each feature by the respective effect size (log odds ratio) derived from the association of the feature with the respective cancer gene class (see Formula 1). Missing continuous features were imputed to the median to facilitate the computation of cancer feature scores for all genes. The correlation between the scores were visualized as a correlation plot (see above).

$$ Score = \mathop \sum \limits_{i = 1}^{n} \beta_{i} \times x_{i} $$

where $\beta_{i}$ is the log odds ratio of association of the nth feature with the respective cancer class and $x_{i}$ is the numeric value of the respective feature. The variation explained by each score was estimated by fitting a logistic regression model with the respective cancer class as the outcome and the score as the exposure. From those models, we report the Nagelkerke pseudo R^2^ measures which denotes the proportion of the variability in the outcome that is explained by model.

### Gene prioritization and pathway enrichment analyses

As a proof-of-principle, we extracted 1250 genes located within 156 loci with genome-wide significant association signals for breast cancer and ranked those genes according to the GWAS cancer score. We recorded the top two ranked genes (high GWAS cancer score set,) as well as all remaining genes (low GWAS cancer score set). We then performed pathway enrichment analyses on both gene sets separately with the *gprofiler2* package (version 0.1.4), as implemented in R using standard settings. Similarly, we performed pathway enrichment analyses in 1640 high- or low-ranking genes in 147 loci associated with coronary artery disease (CAD). We only considered pathways with a maximum term size of 1000 genes and a precision of at least 2.5% (i.e. at least 2.5% of all genes in either gene set need to map to the respective pathway).

In addition, we investigated the enrichment of pathways using a gene set enrichment algorithm implemented in *Webgestalt* 2019 (www.webgestalt.org^30^. We submitted the list of breast cancer genes ranked by the GWAS cancer score to Webgestalt. The gene set enrichment analysis was performed with standard settings but we only considered pathways with a maximum term size of 1000 and a minimum term size of 20.

The pathway definitions were downloaded from MSigDB (URL: https://www.gsea-msigdb.org/gsea/msigdb)^31^. In total, we included 15,922 Gene Ontology Biological Process (GO:BP) pathways, 4582 Gene Ontology Molecular Function (GO:MF) pathways, 50 Hallmark pathways, 186 Kyoto Encyclopedia of Genes and Genomes (KEGG) pathways, 2186 Reactome pathways (REAC) and 521 Wiki Pathways (WP).

## Results

**Figure 1:**
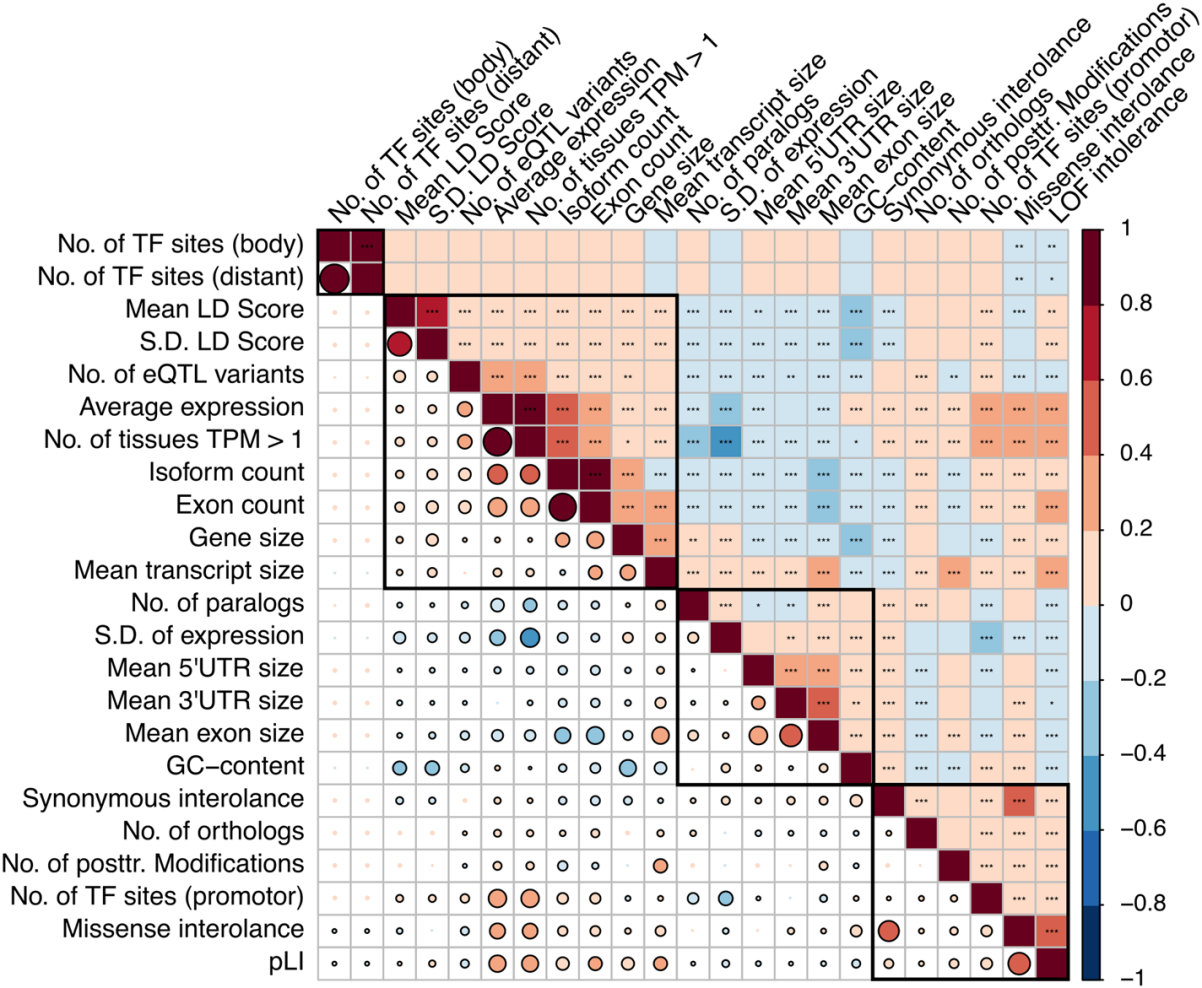
Correlation between *omic* features. 23 *Omic* features of 19,271 protein coding genes were extracted from multiple databases and the pairwise correlation was computed. The features were ordered and four distinct clusters were highlighted according to the hierarchical clustering as implemented in the *corrplot* function. The colour and the size of the circles represent the strength of the correlation (i.e. correlation coefficient) and statistical significance is indicated with asterisks in the upper triangle. Significant correlations (*P* < 0.05) are indicated by a black border in the lower triangle. *TF* transcription factor; *LOF* loss of function; *TPM* transcripts per kilobase million; *S.D* standard deviation *P < 0.05; **P < 0.01; ***P < 0.001. Generated with the *corrplot* function from the *corrplot* package (version 0.84).

First, we established a set of 23 distinct *omic* features from publicly available databases and evaluated the pairwise correlation between those features. The features were broadly clustered into four major groups (Fig. 1) according to their correlation coefficients. As expected, measures of the structure of genes such as size and number of isoforms/exons was highly significantly correlated with another. Similarly, genes which are intolerant towards deleterious mutations are also sensitive to synonymous and missense mutations and generally have fewer paralogs in the genome.

**Figure 2:**
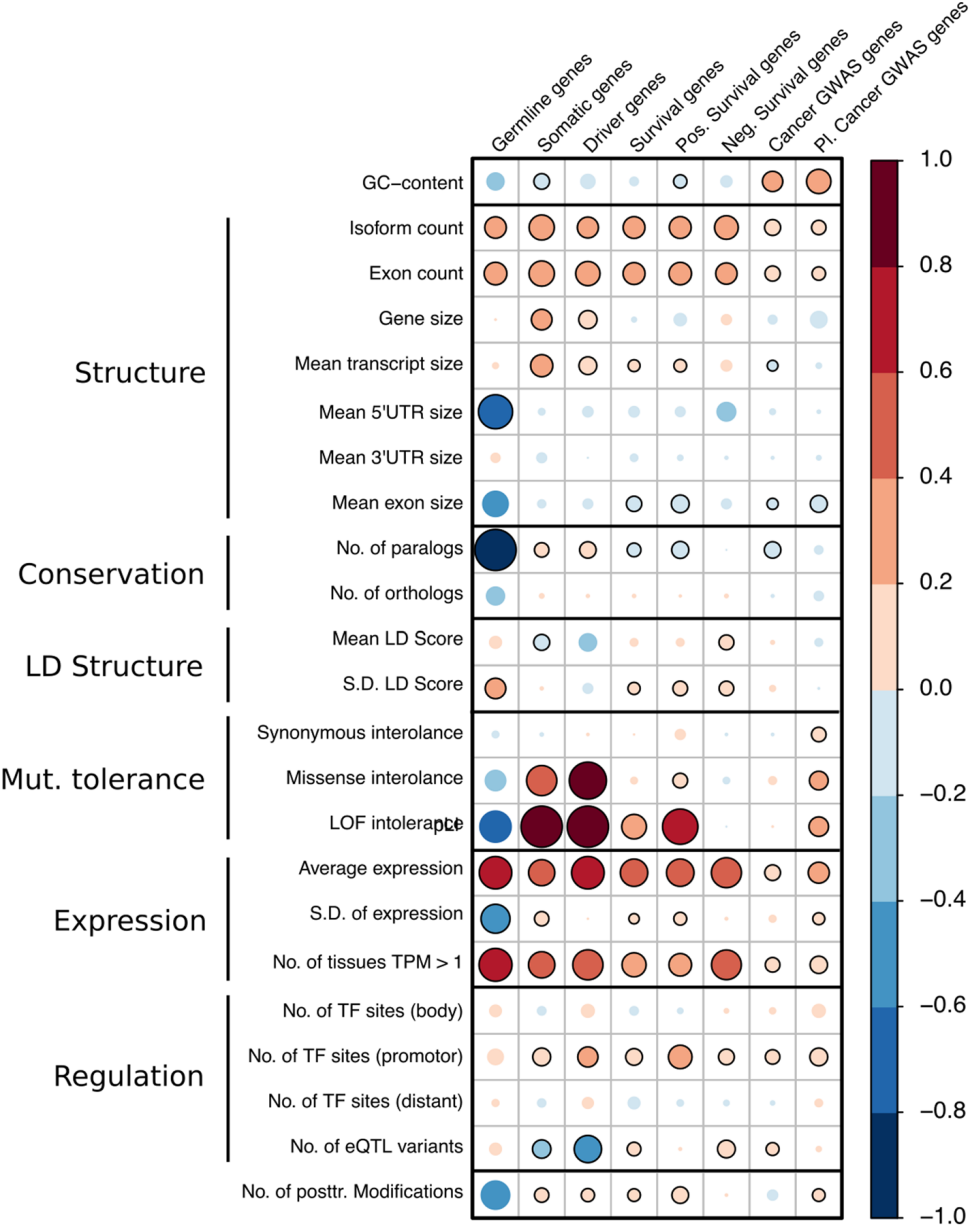
*Omic* features of cancer genes. The size and colour of the circle represent the log odds ratio of the association of 23 *omic* features with different classes of cancer genes compared to non-cancer genes. Associations which were statistically significant (*P* < 0.05) are marked with a black border around the circle. *TF* transcription factor; *LOF* loss of function; *TPM* transcripts per kilobase million. Generated with the *corrplot* function from the *corrplot* package (version 0.84).

Next, we defined 4247 genes to be cancer genes according to expert curated databases as well as published large scale sequencing efforts and genome-wide association study results and grouped them into six cancer gene classes. In total, we included 43 genes which are often harbouring rare cancer predisposition mutations (germline genes), 457 genes frequently mutated in tumours (somatic genes), 106 cancer driver genes as identified by Bailey et al. 2018^2^ (driver genes), 1268 genes whose expression levels are associated with cancer mortality (survival genes) as well as 2373 genes located in cancer GWAS loci (GWAS cancer genes, 901 pleiotropic and 1472 non-pleiotropic). We also established a non-cancer background gene set consisting of 14,110 genes. We evaluated the association of 23 *omic* features (Fig. 1) with cancer gene status and found multiple statistically significant correlations (Fig. 2 and Supplementary Table 1). In general, compared to the non-cancer genes, cancer genes are longer and have more isoforms and therefore are characterized by more numerous and shorter exons. While the GC content of the gene body of cancer genes seems to be lower than in the GC content observed in control genes, we observed the opposite effect for cancer genes found within GWAS loci. Interestingly, cancer genes seem to have fewer paralogs within the genome and as such are expected to be less tolerant towards deleterious mutations. Indeed, our results confirm that most types of cancer genes are indeed more intolerant towards missense and loss-of-function mutations. However, we found no such effect for germline genes (Fig. 2).

Furthermore, we observed that cancer genes are likely not located on longer haplotypes since variants in the gene body do not have a higher average LD Score than control genes. However, we observed an increased variation of the LD Score of variants within the gene body of cancer genes, indicating that those regions harbour a larger diversity of differently sized haplotypes.

The average expression as well as the number of genes expressed across 52 tissues from the GTEx project was significantly increased in cancer genes, highlighting their importance for homeostasis in multiple tissues. As such, cancer genes also harbour more transcription factor binding sites within the promotor across most transcription factor classes (Fig. 2). However, the spread/variation of expression across all GTEx tissues was lower for germline genes, indicating a more uniform expression throughout the body. Notably, we observed a reduced number of transcription factor binding sites in the gene body and in the distal regions of a gene as well as a reduced number of unique eQTL variants in somatic genes. Therefore, somatic cancer genes seem to be preferentially regulated by their promotor, which may indicate more direct and immediate expression control and less influence of distal regulatory processes. Finally, we also observed that cancer genes in general have more post-translational modification.

**Figure 3:**
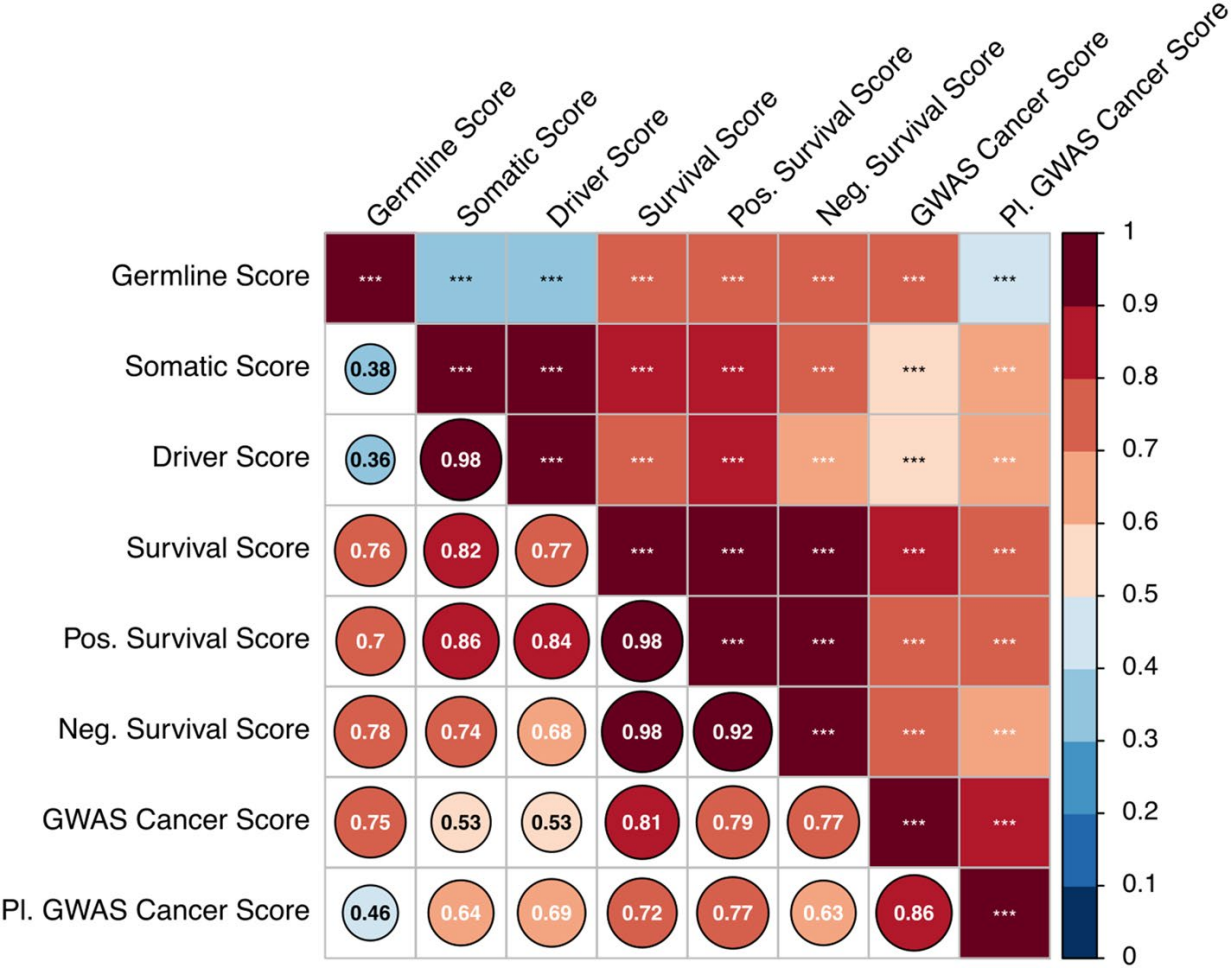
Pairwise correlation between cancer feature scores. For each gene, seven different cancer feature scores were computed by calculating the sum of 23 *omic* features, weighted by the respective log odds ratio estimated from the association with the cancer gene class (see Fig. 2 and Supplementary Table 1). The size and colour as well as the number in the lower triangle represent the correlation coefficient. Statistical significance is indicated with asterisks. *** = *P* < 0.001. Generated with the *corrplot* function from the *corrplot* package (version 0.84).

The identification of features which are able to distinguish between cancer and non-cancer genes as well as between different classes of cancer genes allowed us to compute cancer feature scores for each gene by multiplying the observed effect size (log odds ratio, Fig. 2 and Supplementary Table 1) with the value of the respective feature and calculating the weighted sum. By computing those scores, we effectively create an *omic* feature profile for each gene which summarizes how strongly a gene resembles a typical cancer gene for a given cancer gene class. We found a generally positive pairwise correlation between all score with the strongest correlation observed between somatic and driver cancer genes. In contrast, the correlation was weakest between the scores for germline and somatic as well as driver cancer genes (Fig. 3). Of note, the correlation between the pleiotropic and non-pleiotropic GWAS cancer scores to other scores were similar, although the non-pleiotropic GWAS score was more strongly correlated to the germline score and the pleiotropic GWAS score showed stronger correlation with the somatic and driver score. Thus, genes in pleiotropic GWAS regions seem to be more similar to somatic and driver genes while genes in cancer GWAS regions with an association for a single type of tumour more closely resemble germline genes. The scores explained between 2 and 14% of the observed variation (Nagelkerke pseudo R^2^), implicating that the scores are not completely capturing all molecular properties of cancer genes or that many cancer genes have yet to be identified.

**Figure 4:**
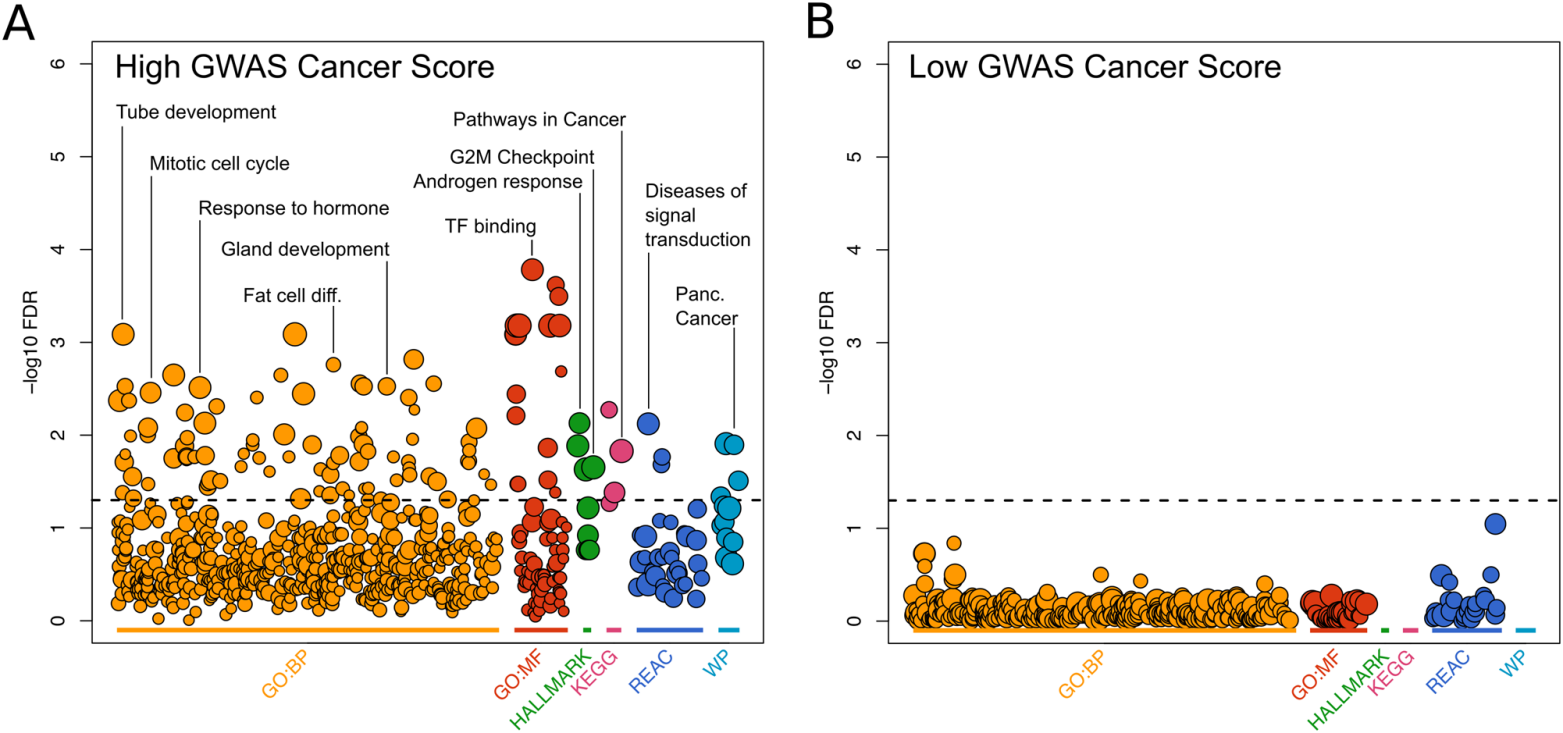
Pathway enrichment analyses for high- and low-ranking breast cancer genes. 1250 genes within 156 breast cancer loci were ranked according to the GWAS Cancer Score (see Fig. 3). Within each locus, the two highest ranking genes as well as the remaining lower ranked genes were extracted. Pathway enrichment for (**A**) high- and (**B**) low-ranking genes were conducted with *gprofiler2* and visualized as a Manhattan plot. Pathways with a Q-value smaller than 0.05 are plotted above the dashed horizontal line and are deemed statistically significantly enriched. *GO:BP* gene ontology biological process; *GO:MF* gene ontology molecular function; *HALLMARK* MSigDB hallmark gene set; *KEGG* KEGG pathways; *REAC* reactome pathways; *WP* wikiPathways; *TF* transcription factor; *Panc.* pancreatic; *diff.* differentiation.

Our GWAS Cancer Score is derived from a pan-cancer approach and thus can potentially be applied to rank candidate genes in a variety of different types of tumours. However, as a proof-of-principle, we chose to prioritize genes in breast cancer loci for potential functional studies, since the number of independent, genome-wide significant signals identified to be associated with breast cancer is among the largest compared to other types of cancer. Therefore, we ranked 1250 genes located within 156 loci with genome-wide significant association signals for breast cancer according to the (non-pleiotropic) GWAS cancer score. We created two gene sets, one including the top two highest-ranking genes within each region (i.e. genes which most closely resemble typical GWAS cancer genes) and another including the remaining genes, which share less similarities with other GWAS cancer genes according to the 23 *omic* features (Supplementary Figure 1). We then performed a pathway enrichment analysis on both, 254 high-ranking and 834 low-ranking genes which mapped to any given pathway (Fig. 4). Importantly, we found no significant enrichment of any investigated pathway in the low cancer score gene set but instead multiple significantly enriched pathways for high ranking genes (Fig. 4). In particular, we found an enrichment for multiple pathways which play a role in cancer initiation and propagation such as pathways related to development, proliferation, cell cycle control, sex hormones as well as transcription factor binding (Supplementary Table 2). To contrast those results to similarly sized gene sets, we investigated the pathway enrichment of 1640 genes (284 high ranking and 1356 low ranking) within 147 loci associated with coronary artery disease^1^. While only the high-ranking gene set of breast cancer GWAS genes showed significantly enriched pathways, we found statistically significant enrichment in both, high- and low-ranking gene sets for coronary artery disease (Supplementary Figure 2). The most significant pathways in both gene sets were related to epithelial cell migration and angiogenesis, transcription factor binding, apoptosis as well as immune response, enzyme inhibition, steroid metabolism and coagulation. Finally, we used a different pathway enrichment algorithm agnostic to our stratification method. To this end, we performed a gene set enrichment analysis on the full breast cancer gene list ranked by the GWAS cancer score (Supplementary Figure 3 and Supplementary Table 3). Similar to the results above, we observed a statistically significant enrichment of pathways relevant to breast cancer in high ranking genes and, conversely, and enrichment of less relevant pathways such as olfactory perception in low ranking genes.

## Discussion

In this study, we have shown that multiple *omic* features are associated with different classes of cancer genes and that cancer genes, in general, occupy a larger region in the genome with a lower GC content, have more isoforms and thus more and shorter exons and are higher expressed across all tissues with more transcription factor binding sites than non-cancer genes. Investigating those features also revealed that germline and somatic as well as driver genes share the least similarities among cancer gene classes and that single tissue cancer GWAS genes more closely resemble germline genes while pleiotropic cancer GWAS genes are more similar to somatic genes. Thus, our approach not only provides novel insights into the molecular properties and differences between cancer gene classes but also allows the prioritization of cancer genes according to their feature profile.

The investigated *omic* features are not completely independent of each other and as such show a varying degree of correlation. While we were able to cluster those features into broadly four groups, the underlying reason for the grouping is not immediately obvious. For instance, in the first large cluster we found that larger genes have more isoforms, are expressed in more tissues with higher levels, are regulated by more eQTL variants and are located on larger haplotypes with more extensive LD. Having more isoforms would allow a gene to have more functions in different tissues and therefore is expected to be expressed at higher levels overall and that this expression is influenced by more eQTL variants, potentially targeting specific isoforms. At the same time, genes with a higher isoform count need to occupy a larger region in the genome in order to fit more alternatively spliced exons within the gene body. Those genes also tend to be under stronger recent positive selection as is evident by their larger extend of LD across the gene body^32,33^ and are more essential due to increased mutation intolerance.

In contrast, genes in the second large cluster are characterized by a higher variation in expression across tissues with more paralogs, a higher GC content in their gene body as well as larger exons and longer 3′ and 5′ UTRs and are more tolerant towards deleterious mutations. Since there are multiple potentially redundant genes present, those genes do not need to rely on multiple isoforms to perform a broader range of functions. As such, they are more likely expressed at high levels in fewer tissues and therefore their variation of expression is larger. Furthermore, their regulation is less guided by eQTL variants but rather by factors binding to the longer UTR regions such as microRNAs or regulatory proteins. Those observations point towards genes which have long been established in the genome and are thus under less positive selection, as is evident by their diminished extend of LD in the gene body.

Multiple omic features showed a high degree of correlation and as such there will be redundancy in the resulting score, potentially amplifying the effect of some features and leading to decreased accuracy or stability of the estimates^34^. However, the germline cancer gene class has only 43 genes and thus approaches such as lasso regression or multivariate logistic regression to extract the most informative features would not be feasible. Nevertheless, we observed that the condition number (the ratio of the largest to the smallest singular values) of the gene × 23 *omic* feature matrix was 12.01 and thus way below 30^35,36^, indicating that the degree of collinearity is not too high to fit multivariate logistic regression models for outcomes (i.e. cancer classes) with more observations. Therefore, we fit a multivariate logistic regression model for GWAS cancer genes with all 23 *omic* features as exposure variables. When we used the resulting effect sizes in the score calculation, we found that this approach only marginally increased the explained variance from 2.4 to 2.9%. This increase is also likely inflated due to the effect sizes being estimated in the same dataset the score was evaluated. Furthermore, the resulting score was highly correlated (R^2^ 0.79) to the score computed from a linear combination of predictors, implicating that our current approach sufficiently captures the underlying molecular properties of cancer genes according to the 23 *omics* features.

Among the most significant finding is the observation that the features of cancer genes are generally similar but also exhibit noteworthy differences. Although all cancer feature scores were positively and significantly correlated with each other, we found the lowest correlation coefficient between germline and somatic as well as driver genes, which can be attributed to multiple observations: while both, germline and somatic genes are expressed at higher levels across all investigated tissues, the variation in expression was markedly reduced for germline and not somatic genes. Therefore, germline genes are expressed at more stable levels since their function in development and maintenance is of importance in almost all tissue types, as previously described^37^. In general, we observed that genes which are more essential (i.e. have a higher LOF intolerance and are thus more intolerant towards mutations) have fewer paralogs thus fewer potential redundant gene copies which would be able to compensate for a potential loss of function in case those paralogues are actually functional and expressed. Whereas somatic genes are indeed less tolerant towards mutations and have fewer paralogs than background genes, we observed an opposite effect in germline genes, highlighting their unique evolutionary path in humans.

By ranking genes within breast cancer GWAS regions, we effectively identified and prioritized a set of genes which share more features with typical GWAS cancer genes. Indeed, pathway enrichment analyses confirmed that the high GWAS gene score set is enriched for multiple processes known or suspected to be involved in breast cancer. Our prioritization approach selected the two top ranking genes within each locus (if present) in order to significantly reduce the number of potential cancer gene candidates for future in-depth characterisation. Alternatively selecting the top 10% ranked genes within each locus resulted in similar enrichment across the regions (data not shown), although the total number of implicated genes within each region varied greatly. We also observed a significant enrichment of breast cancer relevant pathways in a gene set enrichment analysis of the breast cancer genes ranked by the GWAS cancer score, which should be agnostic to specific gene score cut-offs. Those results indicate that different selection and ranking procedures or pathway enrichment procedures should not greatly influence the observed results. Since genes with similar function are often aggregated in the same genomic region it is possible that more than two genes within a given locus are influenced by the associated variants and thus play a role in disease risk. Therefore, for a given locus, the overall distribution of the cancer score should be considered when selecting candidates for further fine-mapping, functional annotation or experimental approaches.

In this study, we used the GWAS cancer score to prioritize breast cancer genes as a proof-of-principle since the number of independent loci is among the largest for breast cancer compared to other types of tumours. While our GWAS cancer score is based on features associated with GWAS genes in any type of cancer, we cannot state with certainty that our approach will work in a similar fashion for other cancer types or for the other classes of cancer genes. Notably, we found that the somatic score is able to explain around 10.5% of the variance of the driver gene class status, probably because they share very similar feature profiles. The driver gene set is based on a pan-cancer approach and includes driver genes from all cancer types included in TCGA, implicating that our algorithm may indeed be applicable in a pan-cancer setting as well.

Our current approach can potentially be augmented by including additional databases with additional and/or more specific features. For instance, aggregating codon level information to gene level from SNVBox^38^ or by downloading bulk gene information via BioThings^39^ may provide increase discriminatory power to distinguish cancer from non-cancer gene. Importantly, our methodology can be applied to other gene sets as well, which can be constructed from genes involved in various diseases or disease groups, from genes located in certain regions of the genome or from genes within specific pathways or processes.

## Conclusion

In conclusion, we have identified multiple *omic* features of different classes of cancer genes, which reveal novel insights into the molecular properties of cancer genes. Those features are generally similar between all investigated classes of cancer genes, although germline and somatic cancer genes share the fewest features, thus potentially reflecting different evolutionary pressure on those two classes of cancer genes. Importantly, the features can be utilized to prioritize candidate genes for future functional studies and may potentially be useful to support (pan-)cancer gene discovery in large-scale sequencing efforts of cancer patients. Our approach can possibly be applied to other gene sets as well to establish unique and shared genomic feature profiles.

## Supplementary information

Supplementary Information 1.

Supplementary Information 2.

Supplementary Information 3.

Supplementary Information 4.

## Data Availability

The data was exclusively retrieved from public repositories and can be accessed from the sources as mentioned in the Methods section. The source code for the computation of the aggregate data will be made available at https://github.com/GrassmannLab/CancerGeneFeatures.
