# Supplementary material for: A systems genomics approach to uncover the molecular properties of cancer genes
Source: Sci Rep. 2020 Oct 27;10:18392. doi: 10.1038/s41598-020-75400-2 (PMC7591476; doi:10.1038/s41598-020-75400-2)
Supplement: Supplementary file 1 — Supplementary Information 1. [file 41598_2020_75400_MOESM1_ESM.docx]

**A systems genomics approach to uncover the molecular properties of cancer genes**

Felix Grassmann^1^, Yudi Pawitan^1^, Kamila Czene^1^

1 - Department of Medical Epidemiology and Biostatistics, Karolinska Institutet, Stockholm, Sweden

**Supplementary Figure 1. Manhattenplot depicting the GWAS Cancer Score of genes in 156 breast cancer loci.** For each gene, the GWAS Cancer Score was computed by calculating the sum of 23 *omic* features, weighted by the respective log odds ratio estimated from the association with the cancer gene class (see **Figure 2** and **Supplementary Table 1**). Genes were ordered according to their chromosomal position. The two genes with the highest GWAS Cancer Score within each locus are highlighted with a black border. The HGNC symbol/name of genes with a GWAS Cancer Score greater than 2.00 are shown above the respective association signal.

**
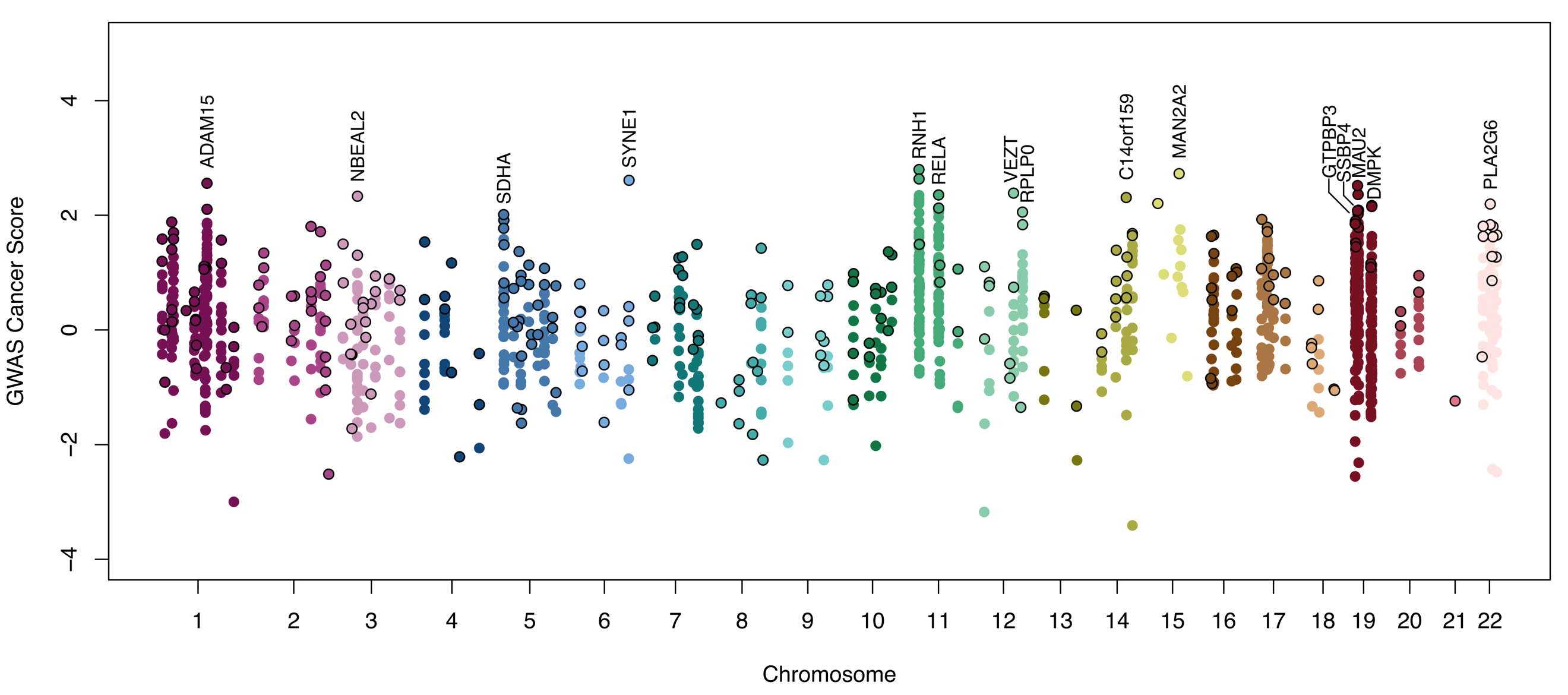
**

**Supplementary Figure 2. Pathway enrichment analyses for high- and low-ranking genes in cardiovascular risk loci**. 1,640 genes within 147 loci associated with coronary artery disease (CAD) ^37^ were ranked according to the GWAS Cancer Score (see **Figure 3**). Within each locus, the two highest ranking genes as well as the remaining lower ranked genes were extracted. Pathway enrichment for (**A**) high- and (**B**) low-ranking genes were conducted with gprofiler2 and visualized as a Manhattan plot. Pathways with a Q-value smaller than 0.05 are plotted above the dashed horizontal line and are deemed statistically significantly enriched. GO:BP=Gene ontology Biological Process; GO:MF=Gene ontology Molecular Function; HALLMARK=MSigDB hallmark gene set; KEGG=KEGG pathways; REAC=Reactome pathways; WP=WikiPathways; TF=transcription factor; Neg.=negative.

**
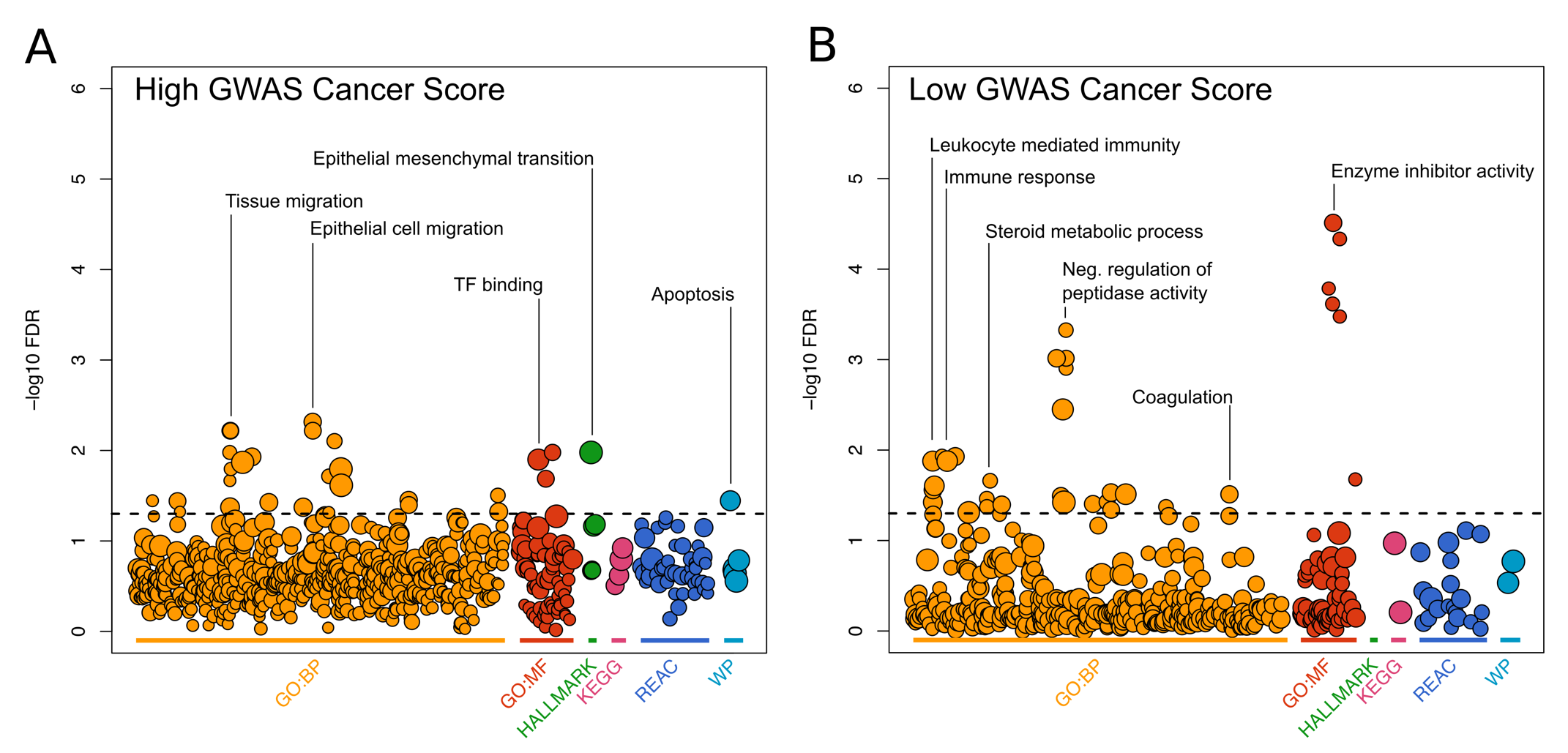
**

**Supplementary Figure 3. Gene set enrichment analysis for breast cancer genes ranked by the GWAS Cancer Score**. Instead of creating two sets, one consisting of genes with either a high or a low GWAS Cancer Score (see **Figure 4**), we ranked all 1,250 genes within 156 breast cancer loci according to the GWAS Cancer Score (see **Figure 3**) and used this ranked gene list in a gene set enrichment analysis as implemented in Webgestalt ([www.webgestalt.org](http://www.webgestalt.org)). Enrichment of pathways within genes with higher scores are indicated with a positive normalized enrichment score while pathways enriched in low ranking genes have a negative enrichment score. Pathways with a statistically significant enrichment (FDR < 0.05) are plotted in full colour, while pathways that did not reach statistical significance are shown as a grey circle. GO:BP=Gene ontology Biological Process; GO:MF=Gene ontology Molecular Function; HALLMARK=MSigDB hallmark gene set; KEGG=KEGG pathways; REAC=Reactome pathways; WP=WikiPathways;


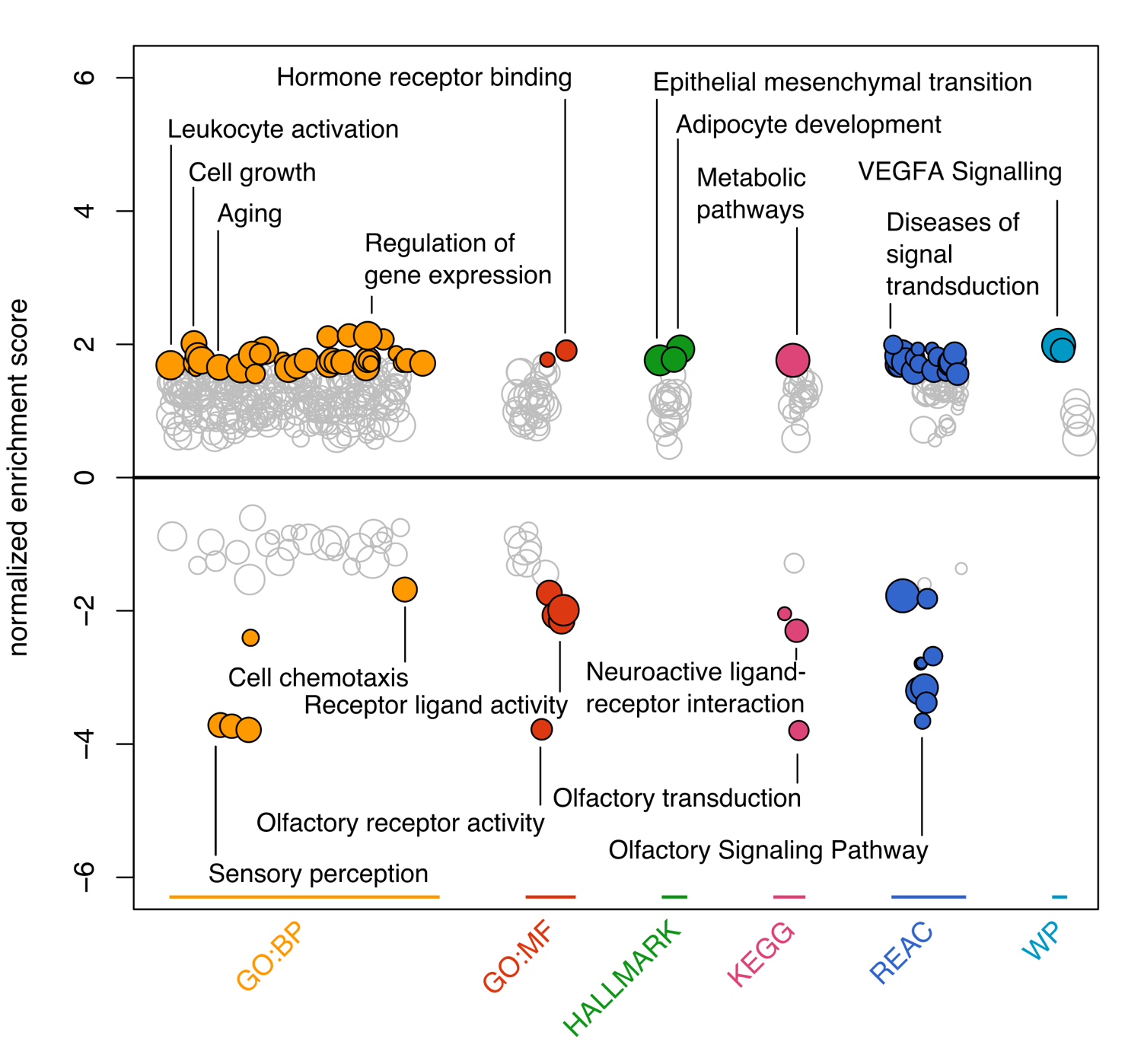


**Supplementary Table 1.** Odds ratio (per S.D.) and 95% confidence intervals of features associated with different cancer gene classes

**Supplementary Table 2.** Pathway enrichment analyses results for high GWAS score genes in BRCA loci.

**Supplementary Table 3.** Gene set enrichment analysis of genes in BRCA loci ranked according to the GWAS Cancer Score.
